# 4397 Virtual Reality Meditation for Acute Post-Operative Pain of Inpatient Adults: Preliminary Results

**DOI:** 10.1017/cts.2020.447

**Published:** 2020-07-29

**Authors:** Nathan J. Dreesmann, Hilaire J. Thompson, Diana T. Buchanan, Hsin Yi (Jean) Tang, Sam R. Sharar, Stephen C. Rayhill, Thomas A. Furness

**Affiliations:** 1 University of Washington; 2 University of Washington, School of Nursing; 3 University of Washington, School of Medicine; 4 University of Washington, College of Engineering

## Abstract

OBJECTIVES/GOALS: This study’s goal is to examine the feasibility and acceptability of using VRM to impact the APP of adults in the inpatient setting. Aims include examining the: 1) feasibility of VRM for APP management; 2) acceptability of using VRM for APP management; and 3) experience of VRM for APP management. METHODS/STUDY POPULATION: To comprehensively examine participants’ experience of using VRM for APP, this study will employ a convergent mixed-methods design in which living kidney donors (N = 45) will be recruited to serially use VRM during their hospital stay. Feasibility and acceptability will be evaluated using descriptive and inferential statistics evaluating patient-reported outcome (PRO) measures taken pre-, post- and 1-hour post-VRM, PRO measures extracted from the participant’s electronic health record and data on VRM use. Semi-structured interviews will allow formulation of inferences based on participants’ experience of VRM for APP management and their insights on content, deployment, and clinical use of VRM. RESULTS/ANTICIPATED RESULTS: This in-process study expects: 1) an adequate sample of participants undergoing living kidney donor surgery who agree to enroll with retention of >90% of participants (Aim 1); 2) participants to report VRM as an acceptable and suitable treatment, feel “present” and interested in the VR environment, and feel comfortable using VRM in the hospital (Aim 2); and 3) to provide insight into participants’ experience of VRM for APP, understanding of extended VRM use for APP analgesia, examination of key variables affecting participants’ experience of VRM for APP and feedback about VRM procedures and protocol to inform future VRM use for APP management (Aim 3). DISCUSSION/SIGNIFICANCE OF IMPACT: Results of the proposed study will inform future clinical testing and deployment of VRM, guide future use of VRM as an adjunct for inpatient APP management, and provide insight into inpatients’ experience of VRM for APP analgesia.

